# Supplementary material for: The causal role of circulating inflammatory markers in osteoporosis: a bidirectional Mendelian randomized study
Source: Front Immunol. 2024 Jul 18;15:1412298. doi: 10.3389/fimmu.2024.1412298 (PMC11291241; doi:10.3389/fimmu.2024.1412298)
Supplement: Supplementary file 1 [file DataSheet_1.zip › supplementary material/OSTEOPOROSIS-CIM/GCST90274801.tsv.gz_leave_one_out.pdf]

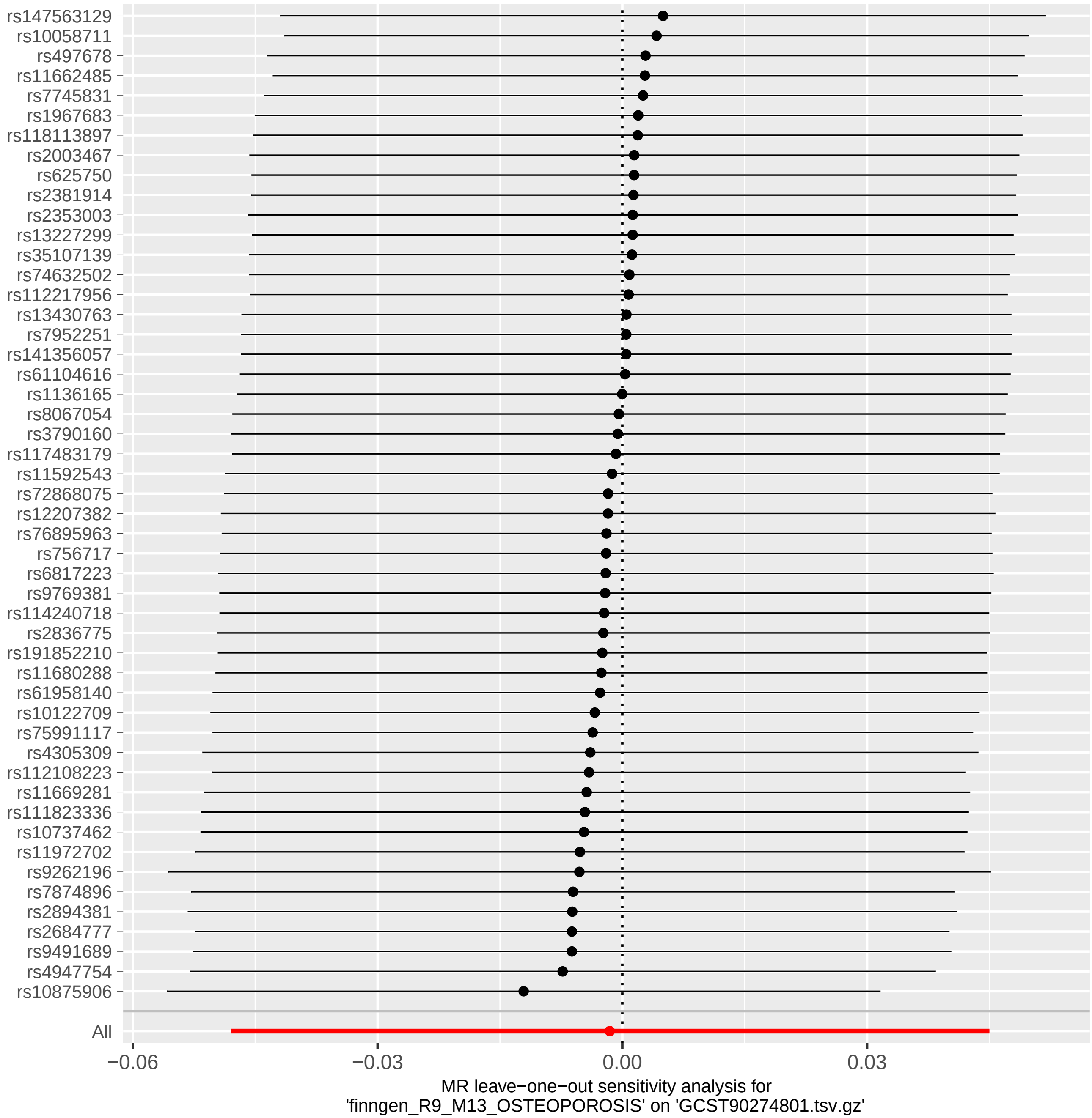
rs147563129
rs10058711
rs497678
rs11662485
rs7745831
rs1967683
rs118113897
rs2003467
rs625750
rs2381914
rs2353003
rs13227299
rs35107139
rs74632502
rs112217956
rs13430763
rs7952251
rs141356057
rs61104616
rs1136165
rs8067054
rs3790160
rs117483179
rs11592543
rs72868075
rs12207382
rs76895963
rs756717
rs6817223
rs9769381
rs114240718
rs2836775
rs191852210
rs11680288
rs61958140
rs10122709
rs75991117
rs4305309
rs112108223
rs11669281
rs111823336
rs10737462
rs11972702
rs9262196
rs7874896
rs2894381
rs2684777
rs9491689
rs4947754
rs10875906
All
−0.06
−0.03
0.00
0.03
MR leave−one−out sensitivity analysis for
'finngen_R9_M13_OSTEOPOROSIS' on 'GCST90274801.tsv.gz'
